# Nutriacción+: A Tool for Learning About Healthy Eating for Economically and Educationally Vulnerable Children

**DOI:** 10.3390/ejihpe15060115

**Published:** 2025-06-19

**Authors:** Diana Arce-Cuesta, Evelyn Pesántez, Pablo Cevallos-Larrea, Cato Van Strijdonck, Michael Peralta

**Affiliations:** 1 Management, Information and Technology Research Group (LABGIT), Salesian Polytechnic University, Cuenca 010102, Ecuador; 2 Biomedical Engineering Research Group (GIIB), Salesian Polytechnic University, Cuenca 010102, Ecuador; evelyn.pesan22@gmail.com (E.P.); pcevallosl@ups.edu.ec (P.C.-L.); michaelperalta1504@gmail.com (M.P.); 3 Faculty of Health and Social Work, Social School, University Colleges Leuven-Limburg (UCLL), 3001 Leuven, Belgium; cato.van.strijdonck@gmail.com

**Keywords:** improve healthy eating knowledge, healthy eating, educational vulnerability, economic vulnerability, iterative cycles, replicable game design process

## Abstract

Ecuador has a high prevalence of malnutrition. In this scenario, Ecuador promotes education on healthy eating through informative materials and talks. However, their content is usually extensive and does not consider the age, economic status, and educational level of the receivers. In addition, children and adolescents often show little interest in learning about healthy eating. In this regard, the literature points to the use of educational games as an effective alternative to improve attention in learning; however, there are few tools addressed to populations in situations of economic and educational vulnerability. This article presents the development and evaluation of an educational game designed to promote learning about healthy eating for children and adolescents in situations of economic and educational vulnerability in Ecuador. The Design Thinking methodology was applied, with three iterative cycles of design, prototyping, and evaluation. Also, a comparative experiment involving 34 participants was conducted to evaluate the observed differences in knowledge acquisition between Nutriacción+ and informative talks. The results analyze how Nutriacción+ contributes to the learning of healthy eating and offer a replicable game design process for similar populations. The results suggest that Nutriacción+ can improve healthy eating knowledge in children aged 8 to 11 years.

## 1. Introduction

Malnutrition represents a global health challenge. According to the World Food Program (WFP), Latin America and the Caribbean face a complex malnutrition problem that includes undernutrition, overweight, and obesity. In that region, between 2000 and 2022, Ecuador has reflected a high prevalence of undernutrition in children (FAO et al., 2023). This type of malnutrition affects the physical and cognitive development of children, increasing their vulnerability to various diseases (FAO et al., 2023; UNICEF, 2021). The analysis “Closing the Nutrient Gap” suggests that the high prevalence of malnutrition in the Ecuadorian population is related to the low level of knowledge about healthy eating practices (Knight et al., 2020). In this context, education on healthy eating is a preceding and necessary step to generate dietary changes in children and adolescents (Chaudhary et al., 2020).

Ecuador has implemented various strategies to address this problem, including the distribution of materials and informative talks to transmit knowledge about healthy eating. However, the content is generally extensive and does not always consider the particularities of age, economic status, and educational level of the different receivers. In addition, children and adolescents commonly show little interest in learning about healthy eating (Yien et al., 2011). In this scenario, Josiemer and Charmaine suggest that nutritional messages should be adapted to the needs and cultural preferences of the population (Josiemer & Charmaine, 2020). Baranowski et al. argue that changing eating habits requires innovative approaches that offer enjoyable experiences to participants while enhancing their ability to make informed decisions about their food choices (Baranowski et al., 2019; Suliyanah et al., 2021; Zafarovna, 2022). In this context, the use of games is an effective alternative to improve children’s attention in educational environments, contributing to the acquisition of knowledge in various fields (Baños et al., 2013; Bell et al., 2018; Chow et al., 2020; Freitas, 2018; Hermans et al., 2018; Lakshman et al., 2010; Selvi & Çoşan, 2018; Suliyanah et al., 2021; Turnin et al., 2001; Yildirim, 2017; Yu et al., 2020; Zafarovna, 2022). Likewise, Contento et al. emphasize that game-based educational strategies should be tailored to the specific motivations and needs of each population group (Contento et al., 1995).

The literature reflects the development and evaluation of educational games for healthy eating (Beber et al., 2024; Bell et al., 2018; Beltran et al., 2012; Johnson et al., 2014; Joyner et al., 2017; Mendonça et al., 2024; Schneider et al., 2012). Such is the case with “Ñami ñam”, a mobile application developed in Ecuador that seeks to promote healthy eating habits in school children (León et al., 2019). “Time to Eat” is a mobile application to promote eating habits in adolescents (Pollak et al., 2010). As well as game proposals aimed at children with intellectual disabilities (Hatzigiannakoglou, 2015; Isasi et al., 2013; Santini et al., 2022). Despite this, limited games are aimed at vulnerable populations. There are no studies focused on the design and use of educational games for the learning of healthy eating for children and adolescents in situations of economic and educational vulnerability. In this sense, educational games can be particularly beneficial for children and adolescents facing social risks, such as poverty, child labor, educational exclusion, and delayed school progression.

This research aimed to develop an educational game, Nutriacción+, to promote healthy eating among economically and educationally vulnerable children and adolescents in Ecuador. The game simulates a shopping environment in popular markets, where many of these children work. Players must buy and exchange food to complete a plate with the highest nutritional value. Nutriacción+ can be played individually or in teams, with an adult moderator guiding the learning and entertainment experience.

The design of Nutriacción+ began with the analysis of the needs of a reference population, composed of children and adolescents in situations of economic and educational vulnerability, together with their educators. All of them are beneficiaries and members of the Salesian PACES Foundation of Ecuador (Fundación Salesiana PACES, n.d.). Subsequently, we involved this population in a process of co-design and evaluation of the game, focusing on aspects of usability and knowledge acquisition.

PACES is a non-profit organization that supports children and adolescents working on the streets and in Cuenca’s popular markets through two care centers. Its beneficiaries, including rural and urban residents, indigenous people, and migrants, receive academic support, food access, and values education.

The following two considerations shaped our hypothesis: (1) the capacity to acquire knowledge about healthy eating can be affected by educational exclusion or delayed school progression, and (2) economically vulnerable children and adolescents often lack access to certain foods and, as a result, are unfamiliar with them. The study hypothesis was defined as follows: the use of Nutriacción+ will increase the participants’ knowledge about healthy eating, in contrast to the use of informative talks, which represent a traditional learning method with general content.

This article presents relevant results along two axes. On the one hand, it discusses how Nutriacción+ could support learning about healthy eating. On the other hand, it describes the Nutriaction+ design process, providing a replicable framework that can serve as a basis for the development of future games targeting similar populations. Bakhtiari et al. suggest that, as most studies focus on why and how games contribute to education, there is a significant need for research that adopts an approach that focuses on the design of educational games. This is to broaden perspectives and encourage innovation within the field of education (Bakhtiari et al., 2024).

This work primarily benefits economically and educationally vulnerable children and adolescents in Ecuador, providing an educational tool tailored to their needs. It also offers support for educators teaching healthy eating to similar populations and expands the understanding of educational games as a resource for teaching vulnerable groups.

## 2. Materials and Methods

We conducted applied and experimental research. The design, development, and evaluation of Nutriacción+ were guided by the Design Thinking methodology. This process focused on understanding the characteristics and specific needs of children and adolescents living in conditions of economic and educational vulnerability.

**Figure 1:**
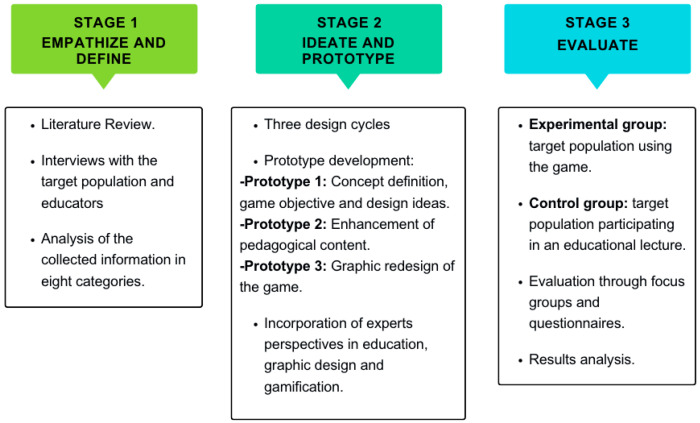
Design and evaluation process of Nutriacción+.

Design Thinking contemplates a non-linear and iterative process to understand users, redefine problems, and create solutions for prototyping and testing (Interaction Design Foundation, 2025). It involves five stages, which are summarized into the following three stages: (1) empathize and define, (2) ideate and prototype, and (3) evaluate, as shown in Figure 1.

### 2.1. Sample Population

Data for this study were collected at the PACES Foundation in the city of Cuenca, Ecuador. Participants were selected from two care centers located in local markets called 9 de Octubre and Feria Libre. Participants in this study included children and adolescents, their parents or legal representatives, and PACES educators. Participants were selected based on the following inclusion and exclusion criteria.

Inclusion criteria are as follows:

■ Children and adolescents between the ages of 4 and 18 years.
■ Active participation in educational or nutrition-related programs at the PACES centers.
■ Availability to attend the game-based activity sessions.

Exclusion criteria:

■ Children with cognitive or communication difficulties that prevented understanding or participation in the activities, as identified by PACES staff.
■ Lack of informed assent from children or consent from their legal representatives.

All participants were recruited through a collaboration agreement with PACES. The recruitment process was facilitated by the foundation’s directors, who provided the necessary institutional approval. To clarify the diversity of children and adolescents’ participants, Table 1 summarizes the demographic data across the different phases of design and evaluation of Nutriacción+.

The children and adolescents who participated in Design Thinking stages 1 and 3 come from urban and suburban areas of Cuenca and belong to low-income households. They are enrolled in primary and secondary schools (Table 1). However, their vulnerability is also shaped by additional factors. Children and adolescents who have experienced domestic violence, including physical, psychological, or sexual abuse, often come from dysfunctional households marked by extreme poverty, parental unemployment, and female-headed households. Children and adolescents who work alongside their parents or relatives in markets or public spaces within Cuenca city typically study in public institutions and participate in PACES programs focused on academic support and personal development. Children and adolescents who engage in activities or work during inappropriate hours or tasks unsuitable for their age, including night shifts, weekends, or long working hours, often either do not attend school or face challenges in their academic performance. Children and adolescents who spend significant time on the streets often experience broken family ties; some have been associated with gang members. Some have a history of legal infractions, come from families with parents deprived of liberty, or have connections to micro-trafficking activities. There are also children and adolescents who are recovering from substance use or who have previously used psychoactive substances (Garzón Vera et al., 2022).

Other participants in this research included PACES educators and the legal representatives of the children and adolescents. The educators consisted of psychology professionals, education specialists, social workers, and culinary experts. The legal representatives had a low educational level. Ethical approval was not required for this study. The project was reviewed by the Research Council of the Universidad Politécnica Salesiana, which certified that ethics committee approval was not necessary due to the educational and minimal-risk nature of the intervention and the absence of sensitive personal data collection. All activities were carried out following ethical research principles. Informed consent was obtained from all participants and, in the case of children and adolescents, from their legal representatives. Participation was voluntary, and data confidentiality and anonymity were strictly maintained.

According to Figure 1, children, adolescents, legal representatives, and educators participated in stage 1 through interviews. Children, adolescents, and educators participated in stage 3 through a usability test and exploratory study where educators served as activity moderators.

### 2.2. Design Thinking: Stage 1—Empathize and Define

The stage began with a literature review to position Nutriacción+ within the context of educational games for healthy eating, education, and vulnerable populations. To understand the profile and needs of future users, semi-structured interviews were conducted with five PACES professionals: two teachers, a chef, a psychologist, and a social worker. These interviews aimed to explore the target population’s economic and educational vulnerability, teaching methods for healthy eating, and associated challenges.

In addition, semi-structured interviews were conducted with 23 child beneficiaries at two PACES care centers. In each center, focus groups were organized based on age. In the first center, 7 participants belonged to the 6–9 years age group, while 5 participants were in the 10–15 years age group. In the second center, 6 participants belonged to the 4–12 years age group, and 5 participants were in the 13–15 years age group.

These interviews identified how children and adolescents interpret healthy eating, what foods they know, their preferences when they buy food on the street, as well as their preferences in recreational activities.

Furthermore, 10 semi-structured interviews were conducted with parents (eight mothers and two fathers) of the participating children to explore eating habits, commonly consumed products, and food access. These findings contributed to the adaptation of the game content, ensuring that it reflected foods familiar and accessible to the study population.

Interviews and focus group activities were audio-recorded, resulting in eight categories: socioeconomic and educational profile, knowledge of healthy eating, eating habits, challenges in adopting good habits, education received, teaching strategies, healthy eating components to reinforce, and family interaction. The analysis aimed to identify patterns and insights to define the game’s learning objectives, key elements, and dynamics that could enhance knowledge acquisition and engagement.

### 2.3. Design Thinking: Stage 2—Ideate and Prototype

Three design cycles were carried out, each of which resulted in the generation of a prototype with its respective evaluation (stage 3). Each evaluation conducted in stage 3 provided modifications to the subsequent prototypes in stage 2, a strategy that corresponds to the non-linear and iterative nature of Design Thinking, according to Figure 1.

#### 2.3.1. First Design Cycle

The objective of this design cycle was to develop a first functional prototype. Based on the empirical evidence collected in the previous Design Thinking stage (Stage 1: Empathize and Define), we conducted a first multidisciplinary design cycle, involving a nutritionist, a Ph.D. in biomedical sciences, a Ph.D. with experience in empirical studies and technology design, and two engineers in biomedical sciences and electronic engineering. The cycle was split into two phases: first, we defined the game’s concept, educational goals, user profile, and integration of healthy eating concepts while discussing possible game features, components, and potential rules for gameplay. In the second phase, we developed a low-fidelity prototype with functional components, denominated Prototype 1. This first design cycle enabled proof of concept, detailed in stage 3.

#### 2.3.2. Second Design Cycle

Once improvements have been implemented according to the results of the proof of concept (stage 3), the objective of the second design cycle was to improve Prototype 1 to subsequently run a usability test. In the second design cycle, four education experts joined the team. In the same way, two phases were executed. In the first phase, they played the game (Prototype 1) to experience its dynamics and usability. Education experts provided feedback on functionality, improvements, solutions, and general aspects via a feedback matrix. The data collected were analyzed to define the necessary changes to Prototype 1. As a result of the second phase, a low-fidelity prototype, denominated Prototype 2, was developed. Prototype 2 strengthened the pedagogical dimension by incorporating an educational guide on healthy eating concepts, an instruction guide, and an electronic roulette. Prototype 2 enabled a usability test detailed in stage 3.

#### 2.3.3. Third Design Cycle

Once improvements have been implemented according to the results of the usability test (stage 3), the objective of this third design cycle was to improve Prototype 2 to subsequently validate the hypothesis raised in this investigation in an exploration context. In the third design cycle, four experts in graphic design and gamification were included. In the first phase of this cycle, they played the game (Prototype 2) to experience its dynamics and usability. Experts analyzed the design of the graphic elements representing food, hygiene habits, and physical activity, as well as the overall environment of the game. As a result, a third high-fidelity prototype, denominated Prototype 3, was developed, incorporating unique graphic elements and a redesign of the typography, logo, and general environment. Prototype 3 was the final prototype of this research and enabled an exploratory study with an experimental approach detailed in stage 3.

### 2.4. Design Thinking: Stage 3—Evaluate

Prototype 1 (stage 2) enabled proof of concept to evaluate the initial game components. Through techniques such as brainstorming and a prioritization matrix, researchers examined the feasibility of the proposal, focusing on the basic functionalities of the game. As a result, adjustments and improvements were made to this prototype.

Prototype 2 (stage 2) enabled a usability test, intending to evaluate user interaction with the game, identify potential design issues, verify functionality, and analyze user acceptance. Qualitative analysis was used to capture the participants’ perspectives on game dynamics, instructions, content, and visual elements. Additionally, it gathered insights into users’ difficulties and their suggestions for improvement. The participants in this test included two PACES educators, as well as children and adolescents experiencing economic and educational vulnerability, as described in the demographic characteristics outlined in Table 1. The usability test was conducted with three distinct groups.

The first group had seven participants (6–14 years), the second had eight (8–16 years), and the third had eight (6–9 years). All groups interacted with Prototype 2 in 40-min sessions.

Researchers observed participant interactions, taking notes of their reactions, comments, and concerns. Subsequently, the children participated in a focus group to discuss their experiences with the game’s usability, including aspects such as dynamics, components, motivation, and suggestions for improvement.

Additionally, semi-structured interviews were conducted to capture the educators’ perceptions of their role as game moderators. They were consulted about the components provided to support their role in terms of being adequate and sufficient and providing suggestions for improvement.

#### 2.4.1. Final Prototype Evaluation (Prototype 3)

The evaluation of the third design cycle (final prototype) represents the formal assessment conducted in this study. The Results section exclusively presents the findings obtained from the final prototype evaluation. This evaluation focused on the following two key evaluation dimensions: (a) Through an exploratory study with an experimental approach, the hypothesis was analyzed, focusing on the observed differences in knowledge acquisition when using Nutriacción+ compared to an educational lecture as a traditional learning method. Specifically, its effect on the increase in participants’ knowledge about healthy eating was evaluated; (b) usability and user experience aspects were evaluated through questionnaires.

##### Hypothesis Exploration

The exploratory study included 34 randomly selected participants: thirty-two (32) children and adolescents with demographic characteristics detailed in Table 1 and two (2) educators as activity moderators. The 32 participants were divided into two iterations, with 8 participants randomly assigned to each of the experimental and the control groups at each iteration. Also, an educator assumed the role of lecturer and moderator of the game, as required.

The interactions took place in three phases. First, a pre-evaluation was applied where a questionnaire was administered to measure the level of knowledge about healthy eating of the children and adolescents before the intervention. Second, the intervention was carried out, being different for the control group and the experimental group. Participants in the control group received an informative talk on healthy eating provided by a PACES educator for 20 min. Participants in the experimental group, for 45 min, used the Nutriacción+ game in a healthy eating learning session. Third, a post-evaluation was immediately applied to measure the change in the level of knowledge after the intervention. Therefore, in this phase, the same questionnaire used in the first phase was administered.

As for data measurement and analysis, the pre-evaluation and post-evaluation questionnaires sought to explore the participants’ knowledge of food classification and basic nutrition concepts. Given the heterogeneity and broad age range of the participants, differentiated questions were applied according to age group. Thus, two subgroups were formed: children aged between 8 and 11 years old and adolescents aged between 12 and 18 years old.

The evaluation tool consisted of two questionnaires tailored for each age group. The questionnaire for children included the following two sections: (1) a set of five true/false statements to assess knowledge of healthy eating habits and (2) three multiple-choice questions addressing everyday practices related to food and hygiene. The questionnaire for adolescents also comprised the following two sections: (1) five multiple-choice questions about nutrition concepts and (2) four true/false statements related to healthy eating and personal hygiene. Higher scores indicate better knowledge and motivation towards healthy eating habits.

The questionnaire items were designed based on guidelines and recommendations from the Food and Agriculture Organization (FAO) (Fautsch Macías & Glasauer, 2014), focusing on key messages in nutrition education for children and adolescents. The instrument was reviewed by two experts in nutrition and education to ensure content relevance, clarity, and age appropriateness. The questionnaire was contextualized for the Ecuadorian population.

The results were analyzed quantitatively using descriptive statistical techniques such as mean, median, standard deviation, and quartiles. In this way, it was possible to perform an analysis by subgroups according to age range, which revealed important differences that were not evident in a general analysis.

The Wilcoxon signed-rank test, a non-parametric method suitable for paired samples, was used to assess changes before and after the intervention. This method was adopted considering the small sample size (*n* = 8) and heterogeneity among participants, particularly in educational background, socioeconomic status, and prior exposure to nutrition-related content, where parametric assumptions such as normality and homogeneity of variance were unlikely to be satisfied. Accordingly, this study emphasizes the identification of preliminary patterns through descriptive statistics, and the inferential findings are interpreted with caution, considering the limited statistical power and generalizability.

##### Exploration of User Experience and Usability

This category included only the 16 children and adolescents of the experimental group. Semi-structured interviews were conducted to assess the game’s usability, experience, and satisfaction. To complement the results, a Likert-scale questionnaire was applied to the educators to capture their general perception of the effectiveness of the game in terms of teaching.

The semi-structured interviews included the following three sections: (a) experience and satisfaction with the game; (b) game dynamics, usability, and difficulties; and (c) improvements and willingness to recommend it. The educators’ questionnaire covered the game’s relevance for children and adolescents in economic and educational vulnerability, its ability to foster interaction and interest, overall satisfaction, and intent to use it in future healthy eating lessons. Finally, results were analyzed qualitatively.

The evaluation results were collected through questionnaires filled out by the participants, as well as audio recordings obtained during interviews and focus group discussions. These audio files were subsequently transcribed to create textual data, which were systematically analyzed to identify key themes.

## 3. Nutriacción+ Game Description

### 3.1. Game Overview

Nutriacción+ is a board game that allows players to differentiate healthy foods from those that are unhealthy or have less nutritional value. The dynamics of the game aim to motivate players to learn about healthy foods and indirectly to change their behavior when buying food.

**Figure 2:**
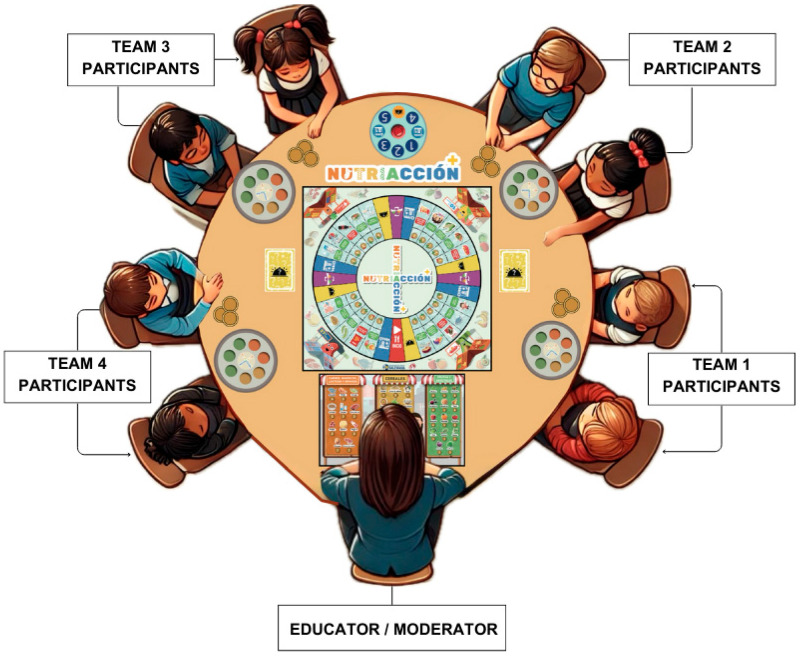
Referential layout of participants and elements in Nutriacción+.

Nutriacción+ is aimed at children and adolescents. The game requires one adult to serve as a game moderator. Figure 2 shows a session of the Nutriacción+ game.

### 3.2. Nutriacción+ Dynamics and Features

**Figure 3:**
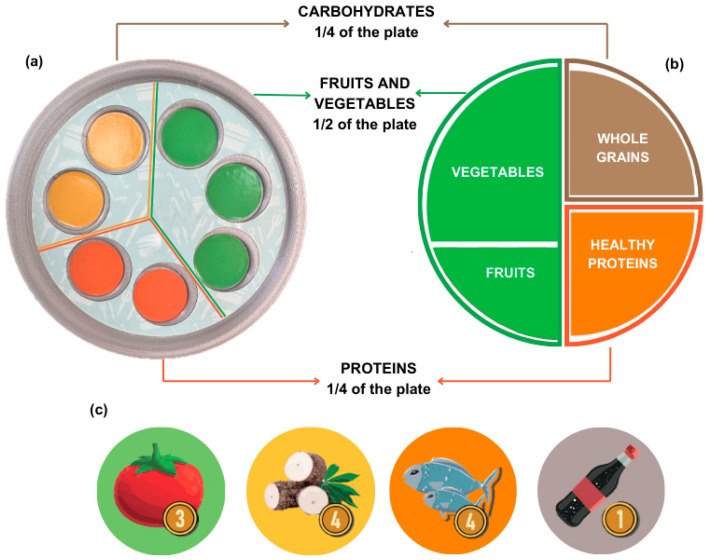
Healthy plate and food pieces: (**a**) Nutriacción+ plate; (**b**) healthy plate; and (**c**) food token pieces examples.

Nutriacción+ recreates popular market spaces where players, individually or working in teams, have the mission of putting together a balanced meal with the maximum nutritional value. The Nutriacción+ plate (Figure 3a) is a functional and educational component that offers a physical space for the incorporation of food in the form of tokens (Figure 3c). It is divided into seven spaces, each identified with a color that provides a visual element to guide and teach players how to combine foods in a balanced way. Its design is based on the Harvard Healthy Eating Plate, Figure 3b (Harvard T.H. Chan, 2012). However, to facilitate interaction, the Nutriacción+ plate simplifies the distribution of food by combining fruits and vegetables in the same group, maintaining the proportions of each of the food groups.

**Figure 4:**
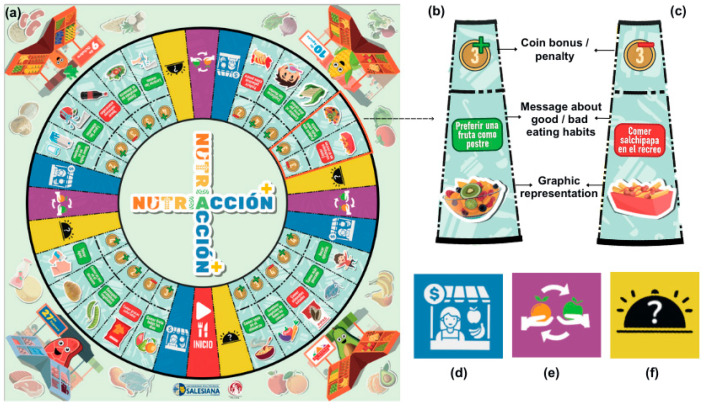
Main board and spaces of Nutriacción+. (**a**) Main board game; (**b**) bonus space; (**c**) penalty space; (**d**) market space; (**e**) trade advantage space; and (**f**) surprise space.

To complete the mission of preparing a healthy plate, the team or player receives an initial number of coins to buy food. Each player (team), represented by a food-shaped player token, must move through 32 spaces on the main board, advancing according to a number obtained from an electronic roulette or a spinning die. During this journey, participants can win or lose coins, buy food at a market stall, or exchange food through bartering in their favor. In addition, participants can face challenges and fun activities that seek to promote interaction. The spaces on the main board (Figure 4a) are classified into four types: bonus (fifteen spaces) and penalty (five spaces); popular market (four spaces); favor trading (three spaces); surprises (four spaces); and start of the game (one space).

Bonus and penalty spaces. The bonus spaces (Figure 4b) show healthy foods or activities. When a player or team lands on this type of space, they win coins given to them by the moderator. The penalty spaces (Figure 4c) show unhealthy foods or activities, so that when a player or team lands on this type of space, they lose coins to the moderator. The moderator, in turn, explains to the participants the reasons behind the addition or subtraction of coins.

**Figure 5:**
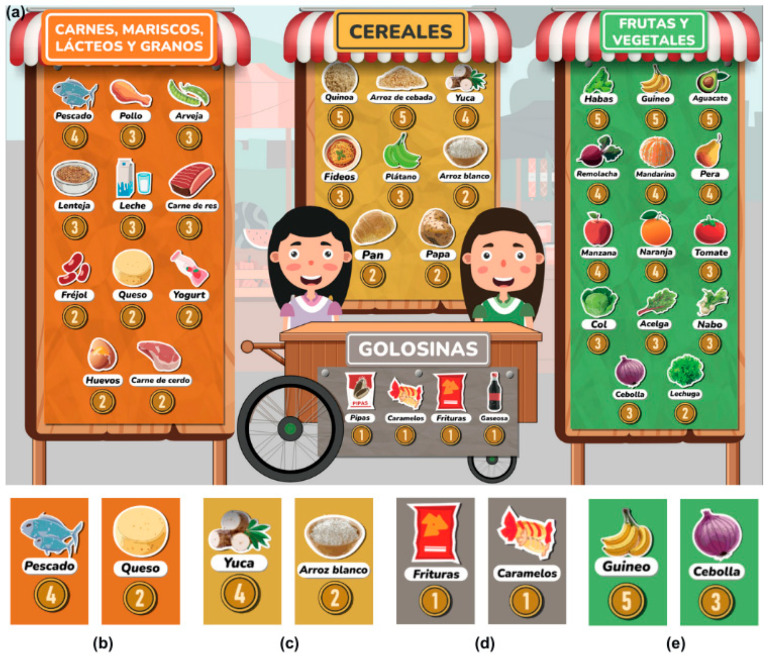
Marketplace board detail. (**a**) Marketplace board; (**b**) protein group; (**c**) carbohydrate group; (**d**) sweets group; and (**e**) fruit and vegetable group.

Popular market spaces. These spaces (Figure 4d) allow participants to buy food from a list presented on a board of products from an external market (Figure 5a). Here, the foods are grouped by color and food category. At this point, the moderator does not intervene in the decision to purchase a particular food but can suggest and present general market information. The purchased food is symbolically delivered to the team/player through a food token piece (Figure 3c). The team will place the token in one of the available spaces on their Nutriacción+ plate (Figure 3a).

Market board. The market board (Figure 5a) displays foods available for purchase, categorized into four groups by color: carbohydrates (yellow), proteins (red), fruits and vegetables (green), and sweets (gray). Foods with higher nutritional value also have a higher economic value. The value system encourages actors to prioritize nutritious foods by linking them to greater economic value. Ecuadorian crops were given higher value to highlight their nutritional importance, as they are often undervalued by local populations.

To identify the nutritional values of each of the foods included on the product board (Figure 5b–e), we used as references the “Table of chemical composition of foods: based on nutrients of interest to the Ecuadorian population” elaborated by the San Francisco de Quito University (Herrera et al., 2021) and the “Food composition table” of the University of Cuenca (Ortiz et al., 2018).

Trade-in favor space. The team or player who occupies this space (Figure 4e) has the option of exchanging food on their plate for one belonging to another player, even if the other player does not agree. The benefiting team can make optional use of this feature, which adds to the competitiveness of the game.

**Figure 6:**
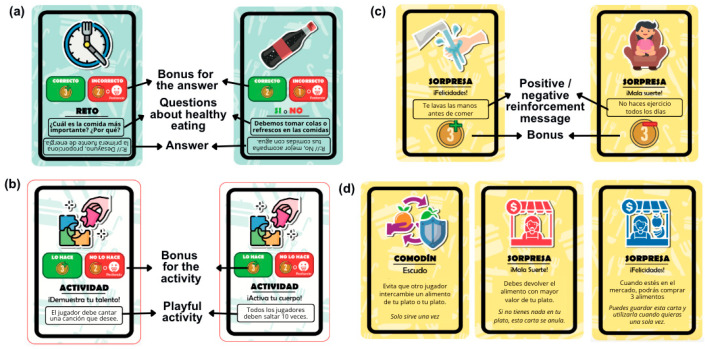
Surprise cards. (**a**) Cards with questions about healthy eating; (**b**) cards with playful activities; (**c**) cards with positive or negative messages; and (**d**) cards that offer strategic advantages or disadvantages.

Surprise cards. In this space (Figure 4f), the team or player randomly receives a surprise card from the moderator. These cards prompt activities like answering healthy eating questions (Figure 6a), performing physical or hygiene tasks, or fun challenges (Figure 6b). Some reinforce learning with positive or negative messages, affecting the game’s economy by awarding or deducting coins (Figure 6c). Nutriacción+ includes wildcard and shield cards (Figure 6d) for strategic advantages, keeping players engaged and making the game fun for active children and adolescents.

The game ends when a team or player creates a plate with the highest nutritional value compared to their competitors’ plates. The plate’s nutritional value is the sum of the values of each food item. The moderator oversees the creation of the plates and determines the winner, also explaining why a particular player or team did not win, helping them understand the impact of their food choices.

**Figure 7:**
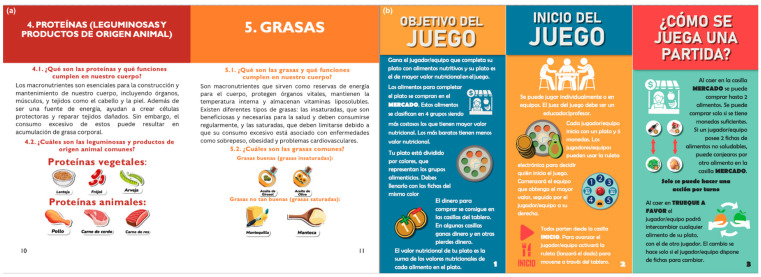
(**a**) Nutritional guide and (**b**) instruction manual for Nutriacción+.

The moderator has the fundamental role of educator, reinforcing and complementing the nutritional information transmitted throughout the game. To this end, Nutriacción+ incorporates a food guide (Figure 7a) as a pedagogical resource to support the moderator.

The development of the food guide (Figure 7a) responded to the need for a moderator trained in healthy eating, as well as the limited availability of nutrition professionals in foundations and educational institutions in Ecuador. The Latin American Alliance for Responsible Nutrition (ALANUR) points out that there is a shortage of nutritionists in Latin America. In Ecuador, there are 5.2 nutritionists per ten thousand inhabitants. This situation affects the regular availability of these professionals to support education in this area (ALANUR, 2023).

Nutrition and biomedicine professionals developed the food guide, considering foods available in Ecuador’s markets and the learning needs of vulnerable children and adolescents. It summarizes key concepts with illustrations on food education and hygiene, organized into the following nine points: (1) healthy eating basics; (2) vitamins and minerals; (3) carbohydrates; (4) proteins; (5) fats; (6) ultra-processed foods; (7) physical activity and hygiene habits; (8) drinking water; and (9) general recommendations.

The moderator profile corresponds to an adult—either a teacher or a parent—who, supported by the food guide, seeks to contribute to the learning of healthy eating habits in their students or children. In addition, the moderator guides the dynamics of the game according to instructions available from Nutriacción+ (Figure 7b) and must enforce the guidelines indicated in the surprise cards (Figure 6).

### 3.3. Technical Specifications of Nutriacción+

**Figure 8:**
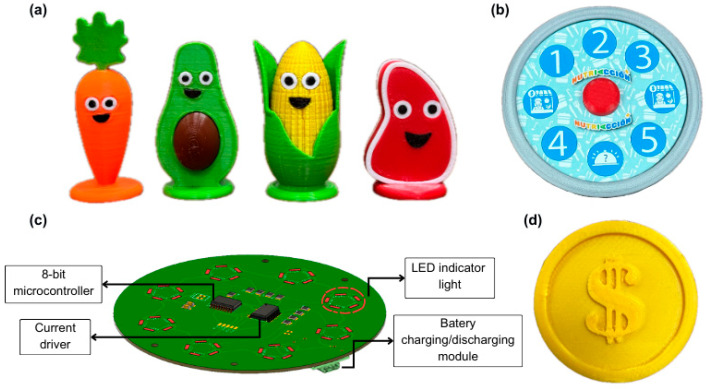
Components of Nutriacción+: (**a**) player tokens, (**b**) electronic spinner, (**c**) electronic circuit of the spinner, and (**d**) coins.

The main game board was printed on a 55 cm × 55 cm sheet of adhesive paper and then mounted on a paperboard base. The back of the board was reinforced with a vinyl sheet. The player pieces were made using 3D printing with low-cost PLA filament (Figure 8a). The coins are the element of exchange (money) in the game (Figure 8d), made in a 2.50 cm diameter and 3D printed.

Another important functional component is the electronic roulette (Figure 8b), incorporated to increase the players’ attention. When activated by a button, it generates a visual sequence of random numbers that indicate how many spaces the player or team advances, also allowing direct access to special spaces such as “Popular Market” or “Surprise”.

The duration of the visual sequence depends on how long the button is held down, and when it ends, the wheel stops at a random position. The roulette circuit (Figure 8c) uses an electronic microcontroller that generates the visual sequence using LEDs and is powered by a lithium battery that can be recharged using its charge/discharge module. The circuit has an integrated circuit that regulates the power consumption of the electronic components. Nutriacción+ also incorporated a spinning die that serves as an alternative to the electronic roulette in cases of low battery or lack of charging facilities.

## 4. Results

### 4.1. Hypothesis Exploration

The hypothesis proposed predicted that the use of Nutriacción+ would increase the participants’ knowledge of healthy eating, in contrast to the use of informative talks, which represent a traditional learning method. Table 2 and Table 3 present the results according to age range, participation group, and type of question.

The results obtained suggest that Nutriacción+ could have a positive effect on increasing knowledge in participants aged between 8 and 11 years. Table 2 shows that playing Nutriacción+ had a positive effect by increasing knowledge scores in the experimental group (mean change: +0.33; *p*-value > 0.05) compared to the control group (mean change: −0.83; *p*-value > 0.05) after listening to a nutrition talk.

Table 3, corresponding to the 12 to 18 age group, presents the indicators of knowledge level categorized into two types: food classification and basic nutrition concepts. According to Table 3, the results suggest that the intervention had a positive effect on the indicator related to food classification, with the control group showing a slight increase (mean change: +0.60), while the experimental group did not show any major changes (mean change: +0.05). Regarding the basic nutrition knowledge indicator, both groups showed an increase after the intervention. The control group improved significantly (mean change: +1.10), while the experimental group showed a moderate increase (mean change: +0.60). All mean change scores (pre-test vs. post-test) obtained no statistical significance for the Wilcoxon signed rank test (*p*-value > 0.05).

### 4.2. Exploration of User Experience and Usability

#### 4.2.1. Experience and Satisfaction When Interacting with Nutriacción+

The participants pointed out that the interaction through Nutriacción+ fostered an environment of trust where they could ask the moderator questions about healthy eating. In addition, there was a preference for playing in teams rather than individually, which can be explained by the fact that Nutriacción+ enables participants to develop joint game strategies and engage in discussions about the ideal food options for their plates. In turn, Nutriacción+ created a familiar and friendly environment for completing the activities proposed on the surprise cards.

However, some differences in perspective were identified between age groups. Participants aged between 8 and 11 years old expressed their pleasure at participating in the activities offered on the surprise cards, while participants aged between 12 and 18 years old generally avoided or showed resistance to carrying out these activities. Adolescents between 12 and 18 years old expressed that the information provided by Nutriacción+ was already known to them, so they did not acquire new knowledge but rather reinforced their previous knowledge. On the contrary, children between 8 and 11 years old expressed that they acquired new knowledge about food classification through the activity of completing a healthy plate.

#### 4.2.2. Usability When Interacting with Nutriacción+

None of the participants had difficulty understanding the information presented on the board and the surprise cards. They also thought that the images of the food represented the food that they knew and ate. The electronic roulette was good for the dynamics of the game and for keeping the participants’ attention.

The participants identified the food purchasing activity in the market as the most engaging part of the game. While the exchange activity (bartering in favor) generated some unfavorable reactions. As stated by the participants, they were initially confused that certain foods of higher cost and nutritional value within the game did not correspond to the cost they are frequently assigned in local markets. However, the dynamics of Nutriacción+ together with the guidance provided by the moderator helped to dispel this confusion.

According to the participants, before starting the game, it is necessary to dedicate some time to an explanation of the rules and components. This is to ensure that all participants understand how it works and can actively participate in it.

#### 4.2.3. Educators’ Perception

Educators who moderated Nutriacción+ found it an effective teaching method that they would use more frequently. One noted it helped to explain healthy eating concepts with greater confidence, while another remained neutral. Both agreed it encouraged children’s participation in discussions and considered it a suitable educational tool. They also observed improved knowledge retention and increased student motivation to learn about healthy eating.

## 5. Discussion

The results of the Nutriacción+ evaluation show signs of its ability to support the acquisition of knowledge about healthy eating. The results, in exploring the hypothesis posed, suggest that Nutriacción+ can be effective in increasing knowledge about healthy eating in children aged between 8 and 11 years. This is relevant in the context of the Ecuadorian population, as studies have identified problems related to malnutrition. A study carried out at the “Jóvenes del Futuro” Foundation in Ecuador reported a prevalence of 65.85% of underweight children aged 5 to 12 (Garcés & Silva, 2024). In an indigenous Ecuadorian community, a prevalence of 22.3% of chronic malnutrition and 21.5% of acute malnutrition was observed (Diaz & de los Zambrano, 2017). Tello et al. point out that 36% of Ecuadorian children aged 5 to 11 are overweight or obese and, at the same time, suggest the need for changes in food education in Ecuador, as well as greater awareness in households (Tello et al., 2024).

### 5.1. Hypothesis Exploration

Descriptive statistics indicate that Nutriacción+ could have a positive impact on knowledge acquisition across both age groups, though the magnitude of this effect appears to vary. While Nutriacción+ shows signs of being effective in teaching healthy eating to children aged 8 to 11, in contrast, adolescents aged 12 to 18 showed a greater increase in knowledge using informative talks. However, the mean score changes in knowledge acquisition showed no statistical significance (*p*-value > 0.05) in both groups.

This may be due to the greater receptivity to change in children, who showed enthusiasm when interacting with Nutriacción+. As well as the potential of board games for learning in children, following studies by Cès et al. (2024). Meanwhile, the adolescents showed less interest in participating and were more easily distracted by constantly checking their cell phones. Based on this, there is a perceived preference among adolescents for digital tools (Marchetti et al., 2015).

Another important consideration in the results is the diversity in participants’ backgrounds. Differences in previous exposure to nutrition-related information (whether through family practices, school experiences, or community programs) may have influenced baseline knowledge and receptiveness to the intervention.

Additionally, the wide age range (8 to 18 years) introduces variability in cognitive development, attention span, and motivation. These age-related differences likely influenced how participants engaged with the educational game and how they retained information, potentially explaining some of the differences observed in the post-intervention results.

### 5.2. Exploration of User Experience and Usability

Positive results were obtained in the qualitative evaluation of usability and user experience. These did not show relevant changes in the components of the game, probably because the evaluated prototype emerged from a process of three design cycles. As a result, this last prototype already incorporated improvements identified in previous evaluations. Despite this, the bartering action in favor was not positively received by the participants, this being a component of attention for future improvements.

On the other hand, participants recommended a detailed explanation of the dynamics of the game before their interaction. Although this aspect was not identified as a critical factor, future improvements should focus on developing mechanisms that facilitate a quick understanding of the instructions to reduce the time spent referring to the instruction guide during the game.

The results reflected the need to design tools that are suitable for the needs and environment of the users. Based on the three design cycles carried out in this research, the following characteristics are suggested for games aimed at children and adolescents in situations of economic and educational vulnerability. (1) The game should be set in a user’s everyday environment; in this study it was a popular market where children and adolescents work. (2) The game should include activities involving interaction and questioning on the subject; in this study it was the choosing and buying of food to teach children to buy healthy food. (3) The game should include team activities that encourage collaboration between children and adolescents and adults. (4) The game should include foods that are known and accessible to children and adolescents in situations of economic vulnerability. (5) The game should include intuitive elements and activities that are understandable and easy to use for children and adolescents in situations of educational vulnerability. (6) The game should be made with wear-resistant materials and components that are easy to replace.

Likewise, the participants indicated that Nutriacción+ favors the participation of children aged 8 to 11 years in contrast to adolescents aged 12 to 18 years. In general, Nutriacción+ promoted, in both age groups, the acquisition of knowledge, teamwork, and greater participation.

### 5.3. Educators’ Perception

The perception of educators was captured as complementary information, as there was a limited number of participants with that profile. The most relevant feedback reflects the educators’ recommendation to use Nutriacción+ as a support tool in the teaching of healthy eating. Therefore, the potential of Nutriacción+ as a teaching tool is an aspect to be studied in future research.

### 5.4. Nutriacción+ Design Process

The design process of Nutriacción+ reflects the integration of educational objectives with design elements according to the characteristics and requirements of a specific population. One aspect to highlight is the interdisciplinary collaboration of professionals in the areas of health, education, and engineering and experts in care for vulnerable populations. This ensured the incorporation of useful components applicable to the context of the children and adolescents who are beneficiaries of PACES.

The iterative design of prototyping and usability evaluation cycles allowed graphic and interaction elements to be adjusted according to the specific needs of children in situations of economic and educational vulnerability. The exploration of the hypothesis as part of the third design cycle of Nutriacción+ allowed for the collection of relevant evidence on the pedagogical objective of the game.

## 6. Conclusions

This research presented the design and evaluation of Nutriacción+, a game for learning about healthy eating aimed at populations in situations of economic and educational vulnerability. Nutriacción+ was developed by a multidisciplinary team, which focused mainly on the characteristics and needs of working street children, beneficiaries of a foundation in the city of Cuenca, Ecuador.

The game design process presented in this article not only contributes to the development of Nutriacción+ but also offers a focus for similar projects aimed at populations in situations of economic and educational vulnerability. The results suggest that Nutriacción+ could have a positive impact on increasing knowledge about healthy eating in children between 8 and 11 years old. However, these results should be interpreted with caution, as they are not conclusive due to the lack of statistical significance.

### Limitations and Future Work

Although the exploration of the hypothesis reflects positive results, these are inconclusive given the limited size of the sample studied, the heterogeneity of the participants, and the lack of statistical significance in the results. Therefore, the validation of the proposed hypothesis requires broader study.

The diversity of participants in terms of age, educational level, economic status, and ethnic origin added complexity to the results. While this increased response variability, it also highlighted age-related differences. These limitations underscore the challenges of designing educational games for vulnerable populations and the need for tailored approaches.

Despite these limitations and challenges, the results of this research provide valuable information on the design and usefulness of Nutriacción+, justifying the need for future research with a larger sample and longer interventions. Thus, in future research, there are plans to conduct a controlled experiment that will expand the sample of this study and analyze in detail the heterogeneity of the participants to homogenize the groups or identify methodologies that better adjust to the diverse characteristics of the population.

Future research could not only evaluate Nutriacción+’s effectiveness in enhancing knowledge of healthy eating but also explore its impact on students’ motivation. Additionally, Nutriacción+ could be studied as a tool to support teachers in teaching healthy eating, with follow-ups to observe long-term changes.

## Figures and Tables

**Table 1:** Demographic characteristics of the children and adolescents participating in the research.

Stage 1 Empathize and Define (Interviews)	Stage 2 Ideate and Prototype	Stage 3 Evaluate (Proof of Concept/Usability Test/Exploratory Study)
Care center “9 de Octubre” 6–15 years old (12 participants; 50% F and 50% M) Geographic location: Urban Ethnic origin: Mestizo Socioeconomic status: Low income. Educational level: Primary and Secondary education. Care center “Ferial Libre” 4–15 years old (11 participants; 63% F and 37% M) Geographic location: Suburban and Urban. Ethnic origin: Indigenous and Mestizo Socioeconomic status: Low income. Educaitonal level: Primary and Secondary education.	First Design Cycle Prototype 1	(Proof of concept) Participation of researchers Children and adolescents did not participate
	Second Design Cycle Prototype 2	(Usability test) First group—Care Center “9 de Octubre” 6–14 years old (7 participants; 43% F and 57% M) Second group—Care Center “Feria Libre” 6–9 years old (8 participants; 62% F and 38% M) Thrid group—Care Center “Feria Libre” 8–16 years old (8 participants; 62% F and 38% M)
	Third Design Cycle Prototype 3	(Exploratory study—Hypothesis) Two care centers (“9 de Octubre” and ” Feria Libre”) 32 participants (34% F and 66% M) Group 1: 8–11 years old (16 participants) Group 2: 12–18 years old (16 participants)

Note: The geographic characteristics of the participants indicated during the evaluation phase—geographic location, ethnic origin, socioeconomic status, and educational level—are consistent with those reported in phase 1, based on data provided by the corresponding study center.

**Table 2:** Results of the exploratory study with the experimental approach—age range between 8 and 11 years.

Group	*n*	Age Range	Moment	Mean (σ)	1st–3rd Quartile
Control	8	8–11 years	Pre-test	5.83 (0.75)	5.25–6.00
			Post-test	5.00 (1.10)	5.00–5.75
Experimental	8	8–11 years	Pre-test	5.50 (1.05)	5.00–6.00
			Post-test	5.83 (0.75)	5.25–6.00

Note: The results correspond to the assessment of “food classification and basic nutrition concepts” in children aged 8 to 11 years. The questionnaire included eight questions: five true/false items with visual cues and three multiple-choice questions. Higher scores indicate better understanding of healthy eating practices.

**Table 3:** Results of the exploratory study with the experimental approach—age range between 12 and 18 years.

Group	*n*	Age Range	Category Questions	Moment	Mean (σ)	1st–3rd Quartile
Control	8	12–18 years	Food classification	Pre-test Post-test	1.80 (0.79) 2.40 (0.70)	1.00–2.00 2.00–3.00
			Basic nutrition concepts	Pre-test Post-test	1.80 (0.92) 2.90 (1.10)	1.25–2.00 2.00–4.00
Experimental	8	12–18 years	Food classification	Pre-test Post-test	1.85 (0.94) 1.90 (0.88)	1.25–2.38 2.00–2.00
			Basic nutrition concepts	Pre-test Post-test	1.20 (1.40) 1.80 (1.62)	0.00–1.75 0.25–3.00

Note: The questionnaire included nine questions: five multiple-choice items and four true/false statements. Higher scores in each section indicate better knowledge of healthy eating practices.

## Data Availability

Requests for access to the data supporting the results of this study be directed to the corresponding author via email (darcec@ups.edu.ec).

## References

[B1-ejihpe-15-00115] ALANUR (2023). Déficit de nutricionistas en Latinoamérica y los retos de enfrentan para combatir las deficiencias alimentarias en la población.

[B2-ejihpe-15-00115] Bakhtiari R., Seraji F., Farrokhnia M., Habibzadeh Z., Noroozi O. (2024). Games in education: A systematic review of studies in international and Iranian contexts. Educational Technology Research and Development.

[B3-ejihpe-15-00115] Baños R. M., Cebolla A., Oliver E., Alcañiz M., Botella C. (2013). Efficacy and acceptability of an Internet platform to improve the learning of nutritional knowledge in children: The ETIOBE mates. Health Education Research.

[B4-ejihpe-15-00115] Baranowski T., Ryan C., Hoyos-Cespedes A., Lu A. S. (2019). Nutrition education and dietary behavior change games: A scoping review. Games for Health Journal.

[B5-ejihpe-15-00115] Beber R. B. C., Doviggi Meyer N., Felipetto G. R., Machado M., Santos M. R., Vargas C. L., Benedetti F. J. (2024). Digital educational game “O Jardim do Ferro”: A tool for the prevention of iron deficiency anemia in childhood. Games for Health Journal.

[B6-ejihpe-15-00115] Bell B. M., Martinez L., Gotsis M., Lane H. C., Davis J. N., Antunez-Castillo L., Ragusa G., Spruijt-Metz D. (2018). Virtual sprouts: A virtual gardening pilot intervention increases self-efficacy to cook and eat fruits and vegetables in minority youth. Games for Health Journal.

[B7-ejihpe-15-00115] Beltran A., O’Connor T., Hughes S., Baranowski J., Nicklas T. A., Thompson D., Baranowski T. (2012). Alpha test of a videogame to increase children’s vegetable consumption. Games for Health Journal.

[B8-ejihpe-15-00115] Cès P., Duflos M., Tricard E., Jhean-Larose S., Giraudeau C. (2024). Playing board games to increase emotional competencies in school-age children and older people: A systematic review. Leisure Sciences.

[B9-ejihpe-15-00115] Chaudhary A., Sudzina F., Mikkelsen B. E. (2020). Promoting healthy eating among young people—A review of the evidence of the impact of school-based interventions. Nutrients.

[B10-ejihpe-15-00115] Chow C., Riantiningtyas R., Kanstrup M., Papavasileiou M., Liem G., Olsen A. (2020). Can games change children’s eating behavior? A review of gamification and serious games. Food Quality and Preference.

[B11-ejihpe-15-00115] Contento I., Balch G. I., Bronner Y. L., Lytle L. A., Maloney S. K., Olson C. M., Swadener S. S. (1995). The effectiveness of nutrition education and implications for nutrition education policy, programs, and research: A review of research. In Database of abstracts of reviews of effects (DARE): Quality-assessed reviews [Internet].

[B12-ejihpe-15-00115] Diaz C. I. E., de los Zambrano A. Á. M. (2017). Estado nutricional en niños de 5 a 11 años de edad en las comunidades indígenas Kumpas y Cumbatza.

[B13-ejihpe-15-00115] FAO, IFAD, PAHO, UNICEF, WFP (2023). Latin America and the Caribbean—Regional overview of food security and nutrition 2023.

[B14-ejihpe-15-00115] Fautsch Macías Y., Glasauer P. (2014). Guidelines for assessing nutrition-related knowledge, attitudes and practices: KAP manual.

[B15-ejihpe-15-00115] Freitas S. (2018). Are games effective learning tools? A review of educational games. Journal of Educational Technology & Society.

[B16-ejihpe-15-00115] Fundación Salesiana PACES (n.d.). Official website of Fundación Salesiana PACES.

[B17-ejihpe-15-00115] Garcés M. F. O., Silva K. J. T. (2024). Prevalencia de la desnutrición infantil en población de 5 a 12 años de edad. Revista Conecta Libertad.

[B18-ejihpe-15-00115] Garzón Vera B., Cárdenas Tapia J., Merchán Arízaga X., López Villavicencio F., Samaniego Sagbay V., Pillco Guamán E., Gordillo Gordillo E., Arce Cuesta D., Jumbo González W. Í., Paucar Paucar J. E., Chávez Barrionuevo J. W., Gallego Condoy M. B., Gallegos Navas M. M., Paredes Floril P. R., Rubio Rubio W. R., Carrera Hidalgo P., Cantuña Ávila A. A., Cañar Tapia C. E., Esquivel Esquivel G. N., Mejía Chafuelan L. B. (2022). Incidencia de los proyectos de Vinculación con la Sociedad de la Universidad Politécnica Salesiana. *Vol. 2*.

[B19-ejihpe-15-00115] Harvard T.H. Chan (2012). Healthy eating plate—The nutrition source. Healthy eating plate.

[B20-ejihpe-15-00115] Hatzigiannakoglou P. (2015). Junk-food destroyer: Helping adolescents with Down syndrome to understand healthy eating through serious game. 2015 7th International Conference on Games and Virtual Worlds for Serious Applications (VS-Games).

[B21-ejihpe-15-00115] Hermans R. C. J., Van Den Broek N., Nederkoorn C., Otten R., Ruiter E. L. M., Johnson-Glenberg M. C. (2018). Feed the alien! The effects of a nutrition instruction game on children’s nutritional knowledge and food intake. Games for Health Journal.

[B22-ejihpe-15-00115] Herrera M., Chisaguano A., Jumbo J., Castro N., Anchundia A. (2021). La tabla de composición química de los alimentos: Basada en nutrientes de interés para la población ecuatoriana|Bitácora Académica.

[B23-ejihpe-15-00115] Interaction Design Foundation (2025). Design thinking. the interaction design foundation.

[B24-ejihpe-15-00115] Isasi A. R., Basterretxea A. L., Zorrilla A. M., Zapirain B. G. (2013). Helping children with intellectual disability to understand healthy eating habits with an iPad-based serious game. 2013 International Conference on Computer Games: AI, Animation, Mobile, Multimedia, Educational and Serious Games (CGAMES).

[B25-ejihpe-15-00115] Johnson M. C., Savio C., Hue H. (2014). “Alien Health”: A nutrition instruction exergame using the kinect sensor. Games for Health Journal.

[B26-ejihpe-15-00115] Josiemer M., Charmaine A. (2020). Strategies for healthy eating promotion and behavioral change perceived as effective by nutrition professionals: A mixed-methods study. Frontiers in Nutrition.

[B27-ejihpe-15-00115] Joyner D., Wengreen H. J., Aguilar S. S., Spruance L. A., Morrill B. A., Madden G. J. (2017). The FIT game III: Reducing the operating expenses of a game-based approach to increasing healthy eating in elementary schools. Games for Health Journal.

[B28-ejihpe-15-00115] Knight F., Mirochnick N., Momcilovic P., Orstavik S., de Pee S. (2020). Cerrando la brecha de nutrientes.

[B29-ejihpe-15-00115] Lakshman R. R., Sharp S. J., Ong K. K., Forouhi N. G. (2010). A novel school-based intervention to improve nutrition knowledge in children: Cluster randomized controlled trial. BMC Public Health.

[B30-ejihpe-15-00115] León B. S., Rodríguez Franco S. A., García León A. (2019). Implementación de un Videojuego Lúdico para el Fomento de Hábitos Saludables para Niños de la Fundación Sonrisa Naranja. Bachelor’s thesis.

[B31-ejihpe-15-00115] Marchetti D., Fraticelli F., Polcini F., Lato R., Pintaudi B., Nicolucci A., Fulcheri M., Mohn A., Chiarelli F., Di Vieste G., Vitacolonna E. (2015). Preventing adolescents’ diabesity: Design, development, and first evaluation of “gustavo in gnam’s planet”. Games for Health Journal.

[B32-ejihpe-15-00115] Mendonça T. S., de Carvalho S. T., Aljafari A., Hosey M. T., Costa L. R. (2024). Oral health education for children: Development of a serious game with a user-centered design approach. Games for Health Journal.

[B33-ejihpe-15-00115] Ortiz J., Astudillo G., Ochoa A., Donoso S. (2018). Tabla de composición de alimentos.

[B34-ejihpe-15-00115] Pollak J., Gay G., Byrne S., Wagner E., Retelny D., Humphreys L. (2010). It’s time to eat! Using mobile games to promote healthy eating. IEEE Pervasive Computing.

[B35-ejihpe-15-00115] Santini F., Tauro G., Mazzali M., Grazioli S., Mauri M., Rosi E., Tarabelloni A., Tizzoni F., Villa F., Molteni M., Nobile M., Sacco M., Aralati S., Colombo V. (2022). A serious game for nutritional education of children and adolescents with neurodevelopmental disorders.

[B36-ejihpe-15-00115] Schneider K. L., Ferrara J., Lance B., Karetas A., Druker S., Panza E., Olendzki B., Andersen V., Pbert L. (2012). Acceptability of an online health videogame to improve diet and physical activity in elementary school students: “Fitter critters”. Games for Health Journal.

[B37-ejihpe-15-00115] Selvi M., Çoşan A. Ö. (2018). The effect of using educational games in teaching kingdoms of living things. Universal Journal of Educational Research.

[B38-ejihpe-15-00115] Suliyanah, Deta U. A., Kurniawan F. K., Lestari N. A., Yantidewi M., Jauhariyah M. N. R., Prahani B. K. (2021). Literature review on the use of educational physics games in improving learning outcomes. Journal of Physics: Conference Series.

[B39-ejihpe-15-00115] Tello B., Ocaña J., García-Zambrano P., Enríque-Moreira B., Dueñas-Espín I. (2024). Determinants of overweight and obesity among children between 5 to 11 years in Ecuador: A secondary analysis from the National Health Survey 2018. PLoS ONE.

[B40-ejihpe-15-00115] Turnin M. C., Tauber M. T., Couvaras O., Jouret B., Bolzonella C., Bourgeois O., Buisson J. C., Fabre D., Cance-Rouzaud A., Tauber J. P., Hanaire-Broutin H. (2001). Evaluation of microcomputer nutritional teaching games in 1876 children at school. Diabetes & Metabolism.

[B41-ejihpe-15-00115] UNICEF (2021). La desnutrición crónica infantil afecta el desarrollo económico y social del Ecuador.

[B42-ejihpe-15-00115] Yien J.-M., Hung C.-M., Hwang G., Lin Y.-C. (2011). A game-based learning approach to improving students’ learning achievements in a nutrition course. Turkish Online Journal of Educational Technology.

[B43-ejihpe-15-00115] Yildirim B. (2017). The effects of educational games, feedback, and correction on the learning level and the retention of knowledge. New Trends and Issues Proceedings on Humanities and Social Sciences.

[B44-ejihpe-15-00115] Yu Z., Gao M., Wang L. (2020). The effect of educational games on learning outcomes, student motivation, engagement and satisfaction. Journal of Educational Computing Research.

[B45-ejihpe-15-00115] Zafarovna E. A. (2022). The role of educational games in English classes. Journal of New Century Innovations.

